# Role of Angular Interface Sign in Characterizing Small Exophytic Renal Masses in Computed Tomography; Prospective Study

**DOI:** 10.15586/jkcvhl.v10i2.262

**Published:** 2023-06-28

**Authors:** Mohamed Sharafeldeen, Mohamed Shaaban, Ahmed Hafez Afif, Mohamed Elsaqa, Nagy Naguib, Sara Elnaggar, Ahmad Beltagy

**Affiliations:** 1 Department of Urology, Faculty of Medicine, Alexandria University. Alexandria Governorate, Egypt;; 2 Department of Radiology, Faculty of Medicine, Alexandria University, Alexandria Governorate, Egypt;; 3 Faculty of Medicine, Alexandria University, Alexandria Governorate, Egypt

**Keywords:** angular interface, computed tomography, ice-cream cone sign, renal masses

## Abstract

The widespread use of computed tomography (CT) has increased the incidence of small renal cell masses. We aimed to evaluate the usefulness of the angular interface sign (ice cream cone sign) to differentiate a broad spectrum of small renal masses using CT. The prospective study included CT images of patients with exophytic renal masses ≤ 4 cm in maximal dimension. The presence or absence of an angular interface of the renal parenchyma with the deep part of the renal mass was assessed. Correlation with the final pathological diagnosis was performed. The study included 116 patients with renal parenchymal masses of a mean (± SD) diameter of 28 (± 8.8) mm and a mean age of 47.7 (±12.8) years. The final diagnosis showed 101 neoplastic masses [66 renal cell carcinomas (RCC), 29 angiomyolipomas (AML), 3 lymphomas, and 3 oncocytomas] and 15 non-neoplastic masses [11 small abscesses, 2 complicated renal cysts, and 2 granulomas]. Angular interface sign was statistically comparable in neoplastic versus non-neoplastic lesions (37.6% versus 13.3%, respectively, P = 0.065). There was a statistically higher incidence of the sign when comparing benign versus malignant neoplastic masses (56.25 vs. 29%, respectively, P = 0.009). Also, comparing the sign in AML versus RCC was statistically significant (52% of AML versus 29% of RCC, P = 0.032). The angular interface sign seems beneficial in predicting the nature of small renal masses. The sign suggests benign rather than malignant small renal masses.

## Introduction

The rate of detection of renal masses has been growing recently due to the widespread use of cross-sectional imaging, including multi-detector computed tomography and novel magnetic resonance imaging sequences. The improvement in spatial and contrast resolutions with imaging has led to more small renal cell carcinomas (RCC) discovered in the earlier stages (stage 1a TNM), which is 4 cm in maximal dimension or less (1,2). Previous studies found that 30% of tumors less than 2 cm in diameter proved benign; on the other hand, 20% of those with a diameter greater than 4 cm were benign (3,4) In addition, the risk of metastatic progression while on active surveillance for small renal masses is low, at <2% in multiple studies (5,6). This has given active surveillance a growing role as a management option for small renal masses, especially in patients with poor performance status (7).

Regarding cystic lesions, the Bosniak classification is now widely accepted and is applied for renal cysts and could help differentiate between benign complex cystic lesions and cystic RCCs (8,9). This mandates other signs and features for more characterization when the small mass is solid or predominantly solid (10).

**Figure 1:**
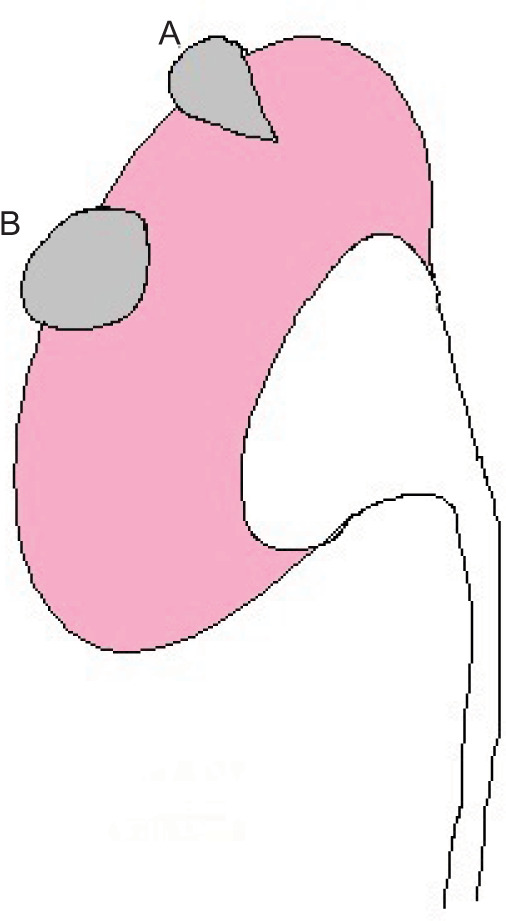
Diagrammatic demonstration of 2 exophytic renal masses; Mass A: shows a positive angular interface sign, and Mass B: has a negative angular interface sign.

Few research of retrospective nature has studied the “angular interface” or “ice-cream cone sign” in the differentiation of benign and malignant small renal masses. As shown in Figure 1, the “angular interface sign” is a radiological sign which describes small exophytic renal masses that have a tapering or pyramidal interface within the parenchyma with a definable apex and exophytic bulge beyond the renal capsule in contrast to other renal masses with rounded interface with the renal parenchyma (negative interface sign) (11,12). Our prospective study aimed to evaluate the usefulness of the “angular interface” sign to differentiate a broad spectrum of small renal masses, including solid and partially solid, inflammatory, and neoplastic masses, using CT.

## Materials and Methods

The study was approved by the institutional review board, and informed consent was obtained from each patient before CT examinations in accordance with the institutional ethics committee. CT images of patients with renal parenchymal masses referred to our radiology department from January 2019 to July 2020 were prospectively assessed. The study included small (≤ 4 cm in maximal dimension), solid, or partially solid (Bosniak IIF, III, IV) parenchymal renal masses with exophytic component of any percentage of the whole mass. Bosniak I and II cysts, completely endophytic lesions, and patients with an unavailable final diagnosis were excluded. Completely endophytic parenchymal masses and masses within the renal collecting system were excluded.

### CT imaging technique

Multi-detector CT examinations were performed on 6-detectors Somatom Emotion 6 (Siemens, Germany), 64-detectors Somatom Perspective (Siemens, Germany), and 16-detectors Brilliance (Philips, The Netherlands). All examinations followed multiphasic renal CT protocol, comprising of non-contrast phase, arterial phase (25 s after the start of contrast injection), venous phase (45 s after the start of contrast injection), nephrographic phase (90–100 s after the start of contrast injection), and delayed phase (10–15 min after the start of contrast injection). Slice-thickness of the source images from each CT machine was 1 mm with no gap.

### Image analysis ad angular interface sign

The source images were reviewed on Osirix 7.0 Lite workstation by two experienced consultant radiologists with 11 and 15 years of experience; both were blinded to each other’s assessment. Image analysis entailed an assessment of the renal masses regarding their nature (solid or partially solid), maximal dimensions in three orthogonal planes, and the presence or absence of angular interface of the renal parenchyma with the deep part of the renal mass (ice-cream cone sign) detected in the phase of the CT examination in which the interface of the renal mass with the renal parenchyma is most conspicuous.

Correlation with the final diagnosis was performed. The final diagnosis was obtained either by pathological data, in case of resected or biopsied masses, or by imaging and clinical data, in cases considered as non-neoplastic, confirmed by follow-up for at least 3 months and up to 12 months. Based on the imaging features and final diagnosis, the “angular interface” sign was statistically evaluated in neoplastic versus non-neoplastic masses, benign versus malignant masses, and angiomyolipoma (AML) versus RCC.

### Statistical analysis

Data were analyzed using IBM SPSS software package version 20.0 (SPSS Inc., Chicago, IL, USA). Qualitative data were described using numbers and percentages. Correlations were calculated using an independent-sample t-test, chi-squared test, and Fisher exact test. P value < of 0.05 was considered statistically significant. Sensitivity, specificity, positive predictive value (PPV), and negative predictive value (NPV) of the “angular interface” sign to differentiate benign from malignant lesions were evaluated. A multivariate logistic regression model including patient’s age, mass side and site, maximal dimension, contrast enhancement, presence of angular interface sign, and presence of intralesional fat was used to evaluate for the features associated with the likelihood of benign versus malignant renal masses.

## Results

The study included CT images of 116 patients with 116 small renal parenchymal masses. Patients were 64 males (55.2%) and 52 females (44.8%), with mean (standard deviation SD) age of 47.7 (±12.8) years. The maximal dimensions ranged from 11 mm to 40 mm with a mean (SD) diameter of 28 (±8.8) mm and a median (interquartile range) diameter of 27 (17-35) mm. Fifty-three (45.7%) masses were in the right kidney, and 63 (54.3%) were in the left. No multifocal or bilateral masses were encountered.

**Figure 2:**
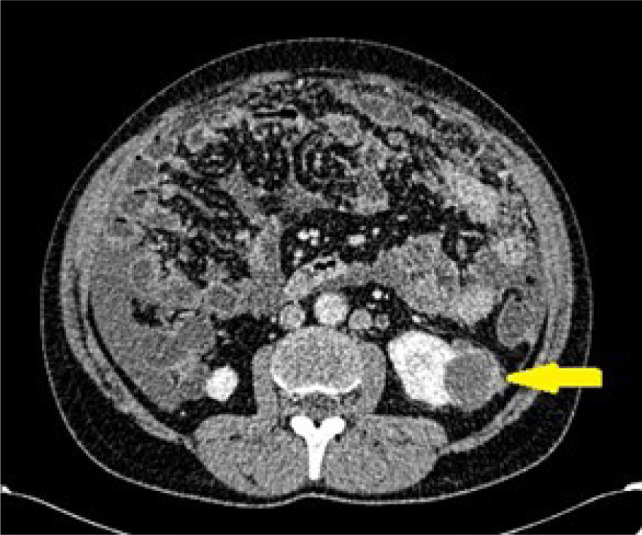
Axial CT at the level of the lower poles of the kidneys in a 43-year-old male showing a 37 mm bi-loculated left renal cyst (yellow arrow) with mild focal enhancing of its wall, categorized as Bosniak IIF cyst, confirmed by no change in size or morphology on 12 months follow-up. The angular interface sign is absent.

After the final pathological diagnosis, 105 neoplastic masses and 15 non-neoplastic masses were included. Table 1 shows the distribution of studied masses according to the final diagnosis. Diagnosis of the 11 abscesses was based upon associated clinical manifestations confirmed by aspiration of pus in 5 abscesses and complete resolution in 3 months, followed by ultrasonography after antibiotic therapy. Two granulomas were diagnosed based upon a history of receiving local BCG treatment for urinary bladder cancer and confirmed by stationary size upon follow-up ultrasonography up to 12 months. Finally, two included complicated renal cysts showed no change in size or texture during a 12-month follow-up by sonography (Figure 2).

**Table 1:** Detailed distribution of the angular interface sign in the non-neoplastic renal masses.

	Pathology (n)		Positive sign	Negative sign
**Non-neoplastic masses**, n (%) *(no= 15)*	Abscesses (11)		2 (18.2%)	9 (81.8%)
	Complicated cyst (2)		0	2 (100%)
	Granuloma (2)		0	2 (100%)
**Neoplastic masses**, n (%) *(no= 101)*	Benign masses *(32)*	Angiomyolipoma (29)	15 (51.7%)	14 (48.3%)
		Oncocytoma (3)	3 (100%)	0
	Malignant masses *(69)*	Clear-cell RCC (42)	4 (9.5%)	38 (90.5%)
		Papillary RCC (23)	14 (60.9%)	9 (39.1%)
		Multilocular cystic RCC (1)	1 (100%)	0
		Lymphoma (3)	1 (33.3%)	2 (66.6%)

Angular interface of the mass with the renal parenchyma “ice-cream cone” sign was generally found in 40 cases (34.5%) and absent in 76 cases (65.5%). The distribution of the “angular interface” sign in the included masses is shown in Table 1.

### Neoplastic versus non-neoplastic masses

The “angular interface” sign was positive in 2 *(13.3%)* non-neoplastic lesions versus 38 *(37.6%)* neoplastic lesions, with no statistically significant difference between both groups (P = 0.065).

### Benign versus malignant neoplastic masses

Neoplastic masses were categorized into benign lesions (no = 32) and malignant lesions (no = 69). The maximal dimension of benign masses was 11–40 mm (mean = 27.7 ± 9.4 mm), while 12–40 mm (mean = 27.7 ± 8.8 mm) for malignant group, P value = 0.92. The analysis of the distribution of the “angular interface” sign in both groups revealed a statistically significant higher incidence of the sign in the benign group (56.25 vs. 29%, p-value of 0.009) with sensitivity, specificity, positive predictive value, and negative predictive value were 56.2%, 71%, 47.4%, and 77.8% respectively (Table 2).

**Table 2:** Distribution of the “angular interface” sign according to the nature of the small renal masses.

	All small renal masses		Neoplastic small renal masses	
	Non-neoplastic masses	Neoplastic masses	Benign masses	Malignant masses
Positive sign	2 *(13.3%)*	38 *(37.6%)*	18 (56.25%)	20 (29%)
Negative sign	13 *(86.7%)*	63 *(62.4%)*	14 (43.75%)	49 (71%)
P value	0.065		0.009	

### AML versus RCC masses

**Figure 3:**
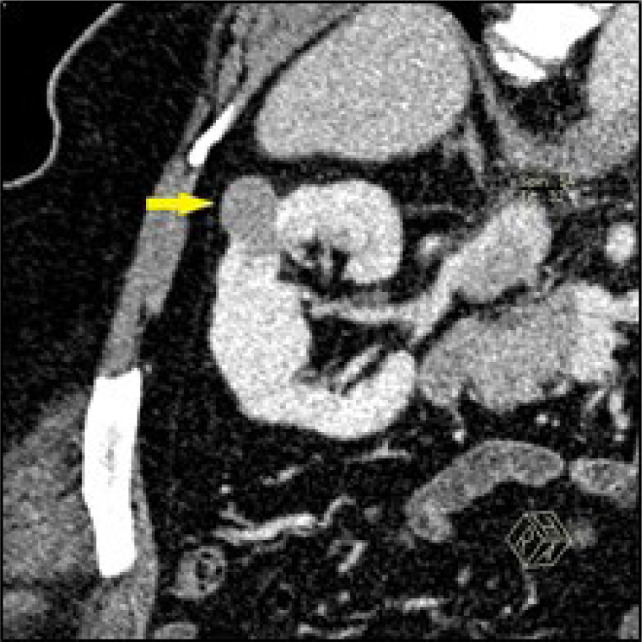
Coronal oblique reformatted CT image of the right kidney in nephrographic phase in a 36-year-old female, showing a 31 mm solid exophytic mass lesion (Arrow) with evident angular interface sign. The mass was enucleated and confirmed lipid-poor angiomyolipoma.

**Figure 4:**
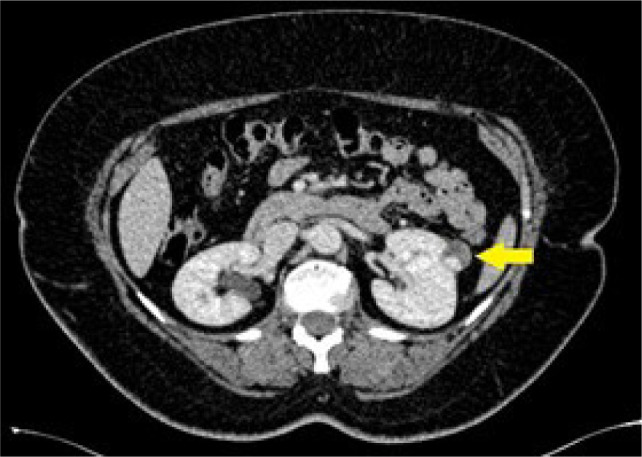
Axial CT at the level of renal hila in a 54-year-old female patient showing a partially solid mass lesion in the left kidney with enhancing small soft tissue component (arrow) and evident angular interface sign. The mass was resected and pathologically showed clear-cell renal cell carcinoma.

The study included 66 cases of RCC and 29 cases of AML with comparable size (mean diameter 27.8 vs. 27.1, 0.73) (Figures 3&4). The “angular interface” sign was present in 15 (52%) AML cases versus 19 (29%) RCC, p-value = 0.032. Sensitivity, specificity, positive, and negative predictive values were 51.7%, 71.2%, 44.1%, and 77.0%, respectively.

Among the 29 AML masses, it was noted that 25 showed gross fat-density (lipid-rich AML) with a positive sign in 11 (44%) of them, while 4 masses were of the lipid-poor type, and all of them showed the “angular interface” sign.

Multivariate regression analysis revealed that masses with smaller size (≤ 2cm versus > 2 cm at maximal dimension, OR = 1.32, 95% CI = 1.16–1.65; p = 0.032), positive angular interface sign (OR = 1.29, 95% CI = 1.19–1.61; p = 0.043) and presence of intralesional fat (OR = 3.56, 95%CI = 1.93–5.52; p = 0.001) were significantly associated with a final diagnosis of benign rather than malignant renal masses.

## Discussion

The pathology of renal masses has a significant impact on management and prognosis. Multiple studies have attempted to differentiate benign from malignant renal masses by imaging by the US, CT, or MRI (13–16). These included studying mass perfusion, signal intensities of the masses in different MRI sequences, and MR Diffusion characteristics of the masses (14,18–23). Dyer et al proposed a strategy to analyze renal masses based on their growth pattern (ball vs. bean). Ball-type masses grow more in an exophytic pattern and deform the renal contour, whereas bean-type masses are infiltrative and may enlarge the kidney (24).

Whereas classical AML is the only benign solid renal mass that can be characterized with confidence by imaging through the detection of bulk fat without calcifications, lipid-poor AML represents a diagnostic challenge. Hindman et al have reported using MRI to differentiate lipid-poor AML from Clear cell RCC. They reported that small-sized lesions and low signal intensity on T2-weighted MR images relative to renal parenchyma suggest lipid-poor AML. At the same time, large size and intra-tumor necrosis favor the diagnosis of RCC (25). Rosenkrantz et al have also studied the MRI features of renal masses measuring < 2 cm, showing that RCC exhibited a higher frequency of T2-hyperintensity, hypervascularity, and cystic/necrotic areas and lower frequency of T2-homogeneity, hemorrhage, and enhancement homogeneity compared to benign renal masses (26).

We prospectively investigated the usefulness of the angular interface “ice-cream cone” sign in differentiating small renal masses, including non-neoplastic, benign neoplastic, and malignant neoplastic masses. Our size criterion was 4 cm or less as a maximal dimension of the renal mass, in concordance with the T1a TNM stage of RCC (27). We evaluated a wide range sample including both solid and partially solid masses, excluding Bosniak I and Bosniak II cystic lesions, as both are readily considered non-neoplastic and require no action in contrast to Bosniak IIF and above, which require at least follow-up (28). The size of renal parenchymal masses showed no statistically significant correlation with the pathology, neither neoplastic versus non-neoplastic nor benign versus malignant neoplastic masses. This matches the same findings reported by previous studies (11,12).

To the best of our knowledge, only two studies have previously described and evaluated the “angular interface” sign in small renal masses; one study was based on CT (11), while the other was based on MRI (12). In contrast to our prospective study, both studies were retrospective. Verma et al (12) investigated the sign in neoplastic lesions using MRI, while Kim et al. (11) compared exclusively lipid-poor AML versus RCC masses, excluding other benign and malignant renal masses using CT. In contrast, our study evaluated the sign in non-neoplastic masses such as small abscesses, granulomas and complicated renal cysts in addition to different neoplastic mass varieties.

In our study, the distribution of the sign did not show a statistically significant difference between neoplastic and non-neoplastic renal masses. The sign was found to be of no use in differentiating neoplastic from non-neoplastic small renal masses. In contrast, the sign helped differentiate benign from malignant neoplastic masses. This matches Verma et al findings (12), who reported a positive sign in 79% of benign complex exophytic masses and 76% of AMLs and a negative sign in all malignant RCC masses using MRI.

Comparing AML versus RCC group, the “angular interface” sign also proved helpful, as the sign was positive in 52% of AML versus 29% of RCC masses. Again this matches with Verma et al and Kim et al findings (11,12). Using CT, Kim et al reported a positive sign in 77.8% of lipid-poor AMLs and 15.6% of RCCs. (11) Through multivariate analysis, Kim et al reported that female gender, small tumor size, and presence of the angular interface sign were predictors of AML versus RCC. It is worth mentioning that the size criteria used by Kim et al differ from ours as they included renal masses up to 3 cm maximal dimension, not 4 cm as in our study (11).

We acknowledge two limitations in our study; the first limitation is that our cases were collected from different CT machines with different detectors rows numbers. However, we tried to overcome this by unifying the slice-thickness of the source images from all CT machines to 1 mm. The second limitation is the low number of lipid-poor AML and oncocytoma which did not allow us to study the usefulness of the “angular interface” sign in differentiating these specific sub-types one from the other.

## Conclusion

The angular interface sign has some utility in predicting the nature of small renal masses. An angular interface sign in the CT imaging of a small renal mass suggests a benign rather than malignant nature.
